# Enhancing marine magnetic anomaly interpretation with anisotropic diffusion and deep transfer learning

**DOI:** 10.1038/s41598-025-30926-1

**Published:** 2025-12-05

**Authors:** J. Ghosh, S. Thoram, Jiajia Sun, W. W. Sager

**Affiliations:** University of Houston, Houston, TX USA

**Keywords:** Mathematics and computing, Ocean sciences, Solid Earth sciences

## Abstract

Linear magnetic anomalies (LMAs) in marine settings provide important clues about formation and evolution of the oceanic crust. LMA interpretation can be challenging as it relies on visual inspection of spatial patterns of magnetic anomalies, which may not be well defined due to sparse and irregular ship tracks. Interpreting such magnetic anomalies based on human perception is inherently subjective as well as time-consuming. We aim to minimize subjectivity and speed up the interpretation by using deep learning (DL). Two significant challenges arise when applying DL to marine magnetic anomalies. First, the anomalies may exhibit discontinuities due to sparse and highly irregular tracklines. Second, the quantity of labeled marine magnetic data maps is very limited. For the first challenge, we employed anisotropic diffusion to smooth LMA along the local orientations, thereby enhancing the continuity. For the second challenge, we investigated deep transfer learning. We implemented three different DL models, namely, standard convolutional neural network (CNN), transfer learning without anisotropic diffusion and with it. When applied to a test dataset consisting of magnetic anomalies from East Pacific Rise and Reykjanes, CNN without transfer learning achieved moderate accuracy. With transfer learning, the prediction accuracy improved substantially. When anisotropically diffused marine magnetic anomalies were used as input, the prediction accuracy reached an even higher level. We applied our best-performing deep learning model—transfer learning combined with anisotropic diffusion—to marine magnetic anomalies from the Shatsky Rise region in the western Pacific and the Azores Plateau region in the northern Atlantic. Predictions at both areas exhibit LMAs resulting from spreading ridge volcanism. Some of the nonlinear predictions are due to poor data coverage, while others are correlated with complex tectonics such as tectonic reorganization, fracture zones, faults, etc.

**Supplementary Information:**

The online version contains supplementary material available at 10.1038/s41598-025-30926-1.

## Introduction

 Marine magnetic anomalies have been pivotal to advance our understanding of Earth’s tectonic processes. Linear and quasi-linear magnetic anomalies are formed by seafloor spreading at mid-ocean ridges^1–3^ when basaltic lava flows cool down and record the direction and intensity of the geomagnetic field. They mark past ridge positions and seafloor isochrons and provide key information about the evolution of oceanic lithosphere and ocean basins. Presence of LMAs indicates a particular style of volcanism – referred to as “ridge volcanism” – in which volcanic emplacement occurs mainly near the ridge axis^3^.

LMAs have been central in advancing the concept of seafloor spreading^1,2^, which ultimately became a key component of plate tectonics. LMA mapped across various ocean basins have provided a record of seafloor ages, spreading rates and directions, as well as plate kinematics, structure, and evolution^4,5^. Marine magnetic anomalies also provide insights into the formation of oceanic plateaus^6^, which are massive underwater volcanic mountains that often rise several kilometers above the surrounding seafloor with areas greater than 10^5^−10^6^ km^2^. These features are located deep underwater and thousands of kilometers away from land, making it difficult to sample these massive oceanic features and collect geoscientific data. Their formation and evolution is still poorly understood.

Marine magnetic data are collected by towing a magnetometer behind a research vessel and most LMAs are identified by visually inspecting the spatial patterns of marine magnetic anomalies. However, interpreting marine magnetic anomalies based on visual inspection is inherently subjective. This is fundamentally due to the fact that marine magnetic anomalies are complex and are influenced by various factors such as faults, fracture zones, tectonics, seamounts as well as sparse and irregular data coverage. Whereas some magnetic anomalies are observed to be unequivocally linear, many others are less so. Moreover, manual LMA identification via visual inspection, for a large area spanning thousands of square kilometers, is tedious and time-consuming.

The goal of our study is to speed up the interpretation of gridded marine magnetic anomalies and to minimize the subjectivity involved in the visual interpretation of such anomalies. To achieve it, we chose DL. Numerous studies have proven that DL excels at learning complex relationships between input and output data, recognizing patterns and automating work processes^7^. DL has been successfully applied across nearly all subdisciplines of Earth science^8–11^, but marine magnetics remains one of the few areas where notable progress has yet to be achieved. Identifying LMAs in a map of gridded marine magnetic anomalies can be thought of as an image classification problem, an area where DL has shown remarkable successes^7,12^. The goal of image classification is to assign a label or category to an input image. When it comes to identifying LMAs, our input is simply a two-dimensional grid of marine magnetic anomalies (for brevity, a “map”). Our objective is to be able to classify such a map into two categories: 0 and 1. Category 0 simply means that the magnetic anomalies in a given map are not dominated by LMAs, whereas Category 1 means they are. This is a classical binary classification problem in machine learning.

Despite its conceptual simplicity and analogy to natural image classification, implementing DL methods for marine magnetic data faces two significant challenges. First, marine magnetic anomalies typically exhibit discontinuities, gaps, and variations in intensity. Some are caused by geological processes but many are due to sparse and highly irregular ship tracklines, in which case the discontinuities are simply artefacts resulting from interpolation of insufficiently and irregularly sampled data in the marine setting. These discontinuities make it harder for humans and, likely, neural networks to identify LMAs that are inadequately sampled by ship tracks. The second challenge results from the lack of a sufficiently large number of training images with labels, also referred to as labeled images in machine learning literature. The success of modern DL methods, to a large extent, is attributed to the availability of massive labeled images. One notable example is ImageNet^13,14^, a publicly available large database with more than 14 million labeled images. ImageNet has been recognized as instrumental in advancing deep learning research. However, a similar database for marine magnetic anomaly interpretation does not exist, which significantly limits the application of modern DL methods to marine magnetics.

To close gaps in anomaly patterns and enhance continuity, we used anisotropic diffusion^15,16^. Anisotropic diffusion is a well-established image processing technique that is often applied to enhance flow-like structures that appear in scientific image processing (ex: flow fields for ocean dynamics), medical imaging, fingerprint analysis etc^16–18^. Diffusion adaptively smooths anomalies based on local gradient information. Different from many other smoothing methods, anisotropic diffusion preserves edges and enhances continuity by effectively inhibiting smoothing across strong edges and promoting it in homogeneous areas and along edges^19–25^. It serves our purpose of enhancing continuity of marine magnetic anomalies.

To address the second challenge associated with the lack of a large set of labeled marine magnetic maps, we resort to transfer learning. Transfer learning is a machine learning technique that enables the reuse of pre-trained models and their learned features as a starting point for a new, but related, task, allowing for faster training and improved performance when only a limited number of labeled images are available^26,27^. In our work, we visually inspected marine magnetic anomaly maps from East Pacific Rise (hereafter referred to as EPR) and Reykjanes Ridge and created 851 labeled images. The size of our labeled images is way too small to properly train a DL model that can correctly identify LMAs in new areas. Transfer learning solves this problem by reusing features^28^ learned based on images from other settings (e.g., tens of millions of labeled images of the natural world in the ImageNet database). In other words, the features learned based on ImageNet are ‘transferred’ to a different domain, in this case, classifying marine magnetic anomaly images.

By combining anisotropic diffusion and transfer learning into a new workflow, we developed a semi-automated method of interpreting marine magnetic anomalies. To the best of our knowledge, this is the first time that deep transfer learning has been used to interpret marine magnetic anomalies. Our new method solves the significant challenges arising from the lack of a large set of labeled training images which is a common issue in many Earth science applications. Compared with traditional human interpretation of marine magnetic anomalies, this new method is much faster and arguably less subjective. We believe that it will greatly assist human experts in interpreting marine magnetic anomalies and gaining new insights into ocean basin geology and tectonics.

## Methods

Anisotropic diffusion and deep transfer learning are two components that distinguish our new methodology from the standard visual inspection and interpretation of marine magnetic anomalies. In this section, we explain what they are and why they are important for our work.

### Anisotropic diffusion

Anisotropic diffusion is an image processing technique that can reduce image noise and enhance image features, such as edges and lines, by adaptively applying the smoothing process based on the local image gradients^15,29^. Unlike isotropic diffusion (e.g., Gaussian blur), which smooths uniformly in all directions, anisotropic diffusion works by adjusting the “diffusion coefficient” at each pixel based on the local image gradient. Where the data values (i.e., marine magnetic anomaly values in our work) are relatively uniform, the diffusion coefficient is high, allowing smoothing within homogeneous regions. Where there is a strong change in data values (e.g., across an edge), the diffusion coefficient is reduced, preserving and enhancing edges. It has been successfully used to denoise MRI or ultrasound medical images to make anatomical features clearer for diagnosis, without blurring the critical boundaries between tissues or organs^30–32^. Fehmers^33^ applied anisotropic diffusion to structural interpretation of 3D seismic data. Smith^34^ developed a constrained anisotropic diffusion method to minimize spatial aliasing in airborne magnetic measurements. We note that the input in Smith^34^ is line data (before gridding), whereas we used gridded marine magnetic maps as input.

**Fig. 1:**
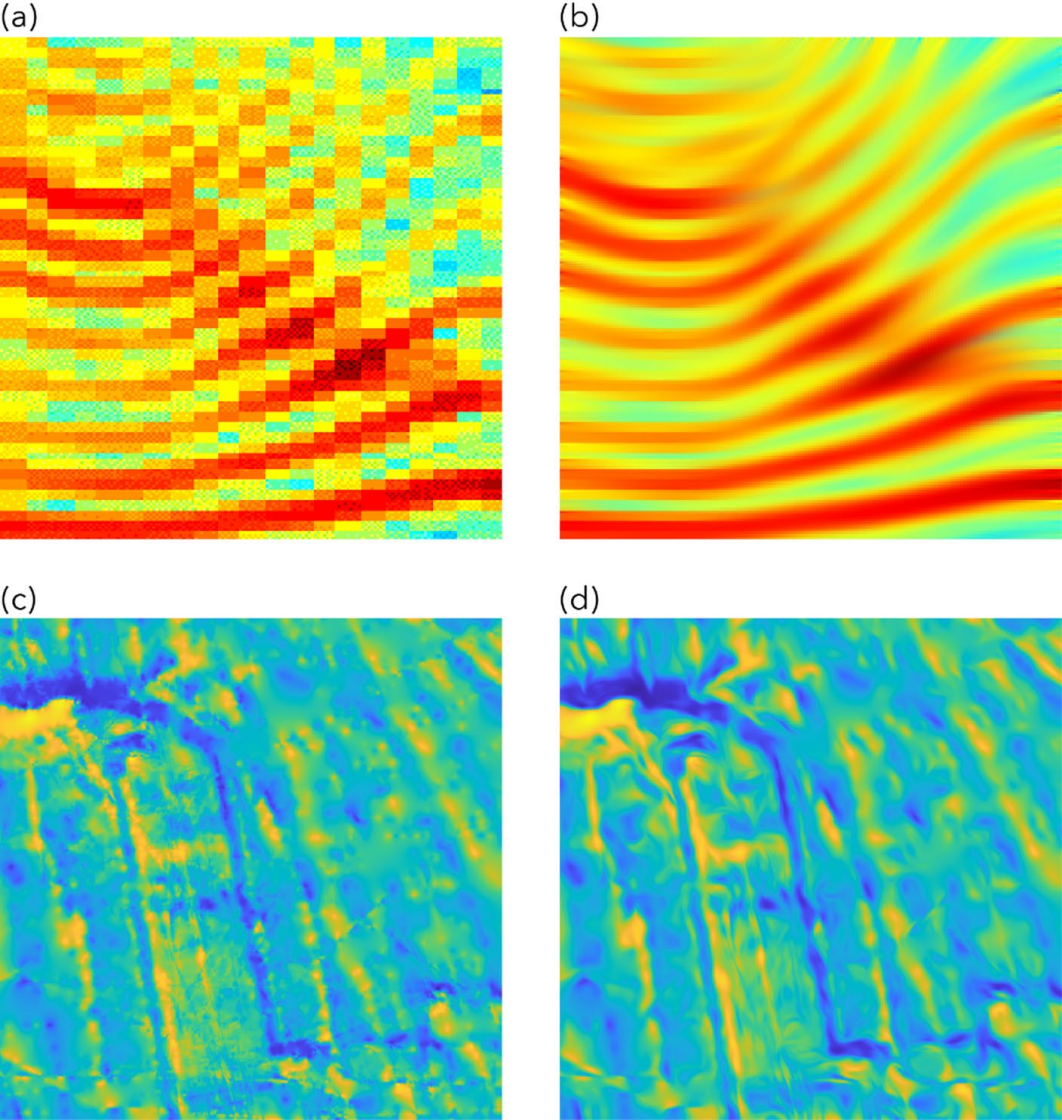
Top: Anisotropic diffusion of a sonogramme (modified after Weickert^16^; (**a**) an intensity plot of local frequency analysis of the Danish word “hej”, in a time (x-axis) vs. logarithm of frequency (y-axis) domain, (**b**) the diffused counterpart of the image in (**a**). Bottom: Anisotropic diffusion of an anomaly map; (**c**) EPR anomaly map before diffusion, (**d**) EPR anomaly map after diffusion.

 Figure 1 shows two examples where anisotropic diffusion is used to enhance the features in two different types of images. A sonogramme is displayed in Fig. 1a. Figure 1b shows the anisotropically diffused sonogramme. We can clearly see that anisotropic diffusion greatly enhances the continuity of the high-energy features. In Fig. 1c, we show some marine magnetic anomalies from the East Pacific Rise region. Due to the sparse and irregular ship tracklines, many of the LMAs in Fig. 1c are disrupted by the lack of measurements. We applied anisotropic diffusion^25^ to the marine magnetic data in Fig. 1c and obtained the diffused image in Fig. 1d. Similar to the sonogramme example, we observe that the overall noise level is greatly reduced, and the diffused magnetic anomalies appear smoother and exhibit a higher degree of continuity. We emphasize that anisotropic diffusion does not ‘create’ continuity out of nothing; instead, it simply enhances it based on the anomaly patterns already existing in the data, as is evident in these two examples. By enhancing the continuity of marine magnetic anomalies, it becomes easier for human experts to identify LMAs. Our hypothesis is that, if it helps humans, it should also help DL models to perform better. Indeed, as discussed in Sect. 4.1, anisotropic diffusion helps further improve the prediction accuracy.

### Deep learning

**Fig. 2:**
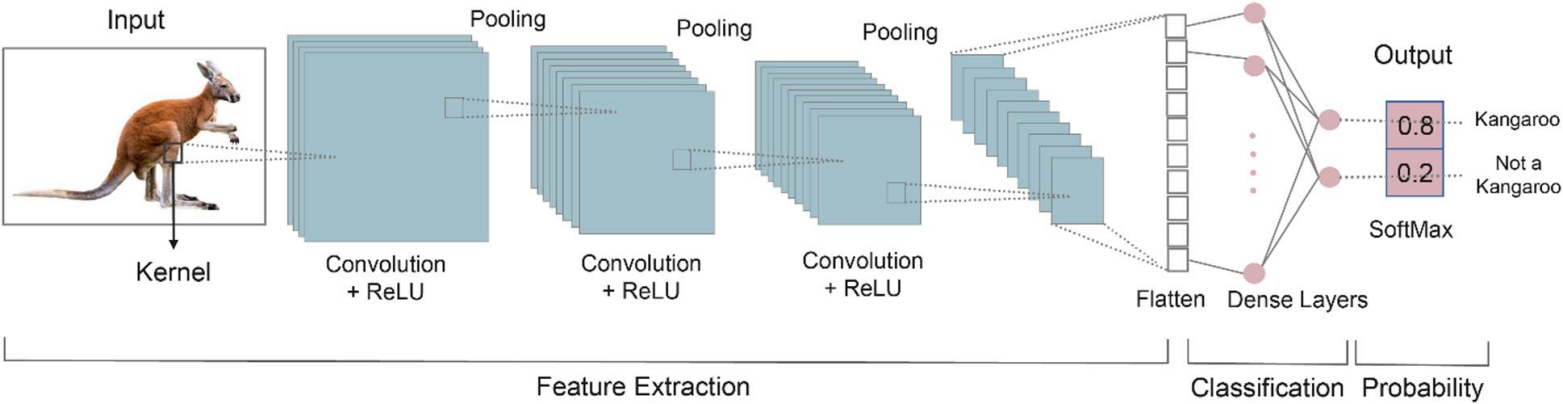
An example of CNN architecture. The input is a 2D data map. The convolutional and pooling layers extract meaningful features from the input map to make correct predictions. The output is a decimal prediction between 0 and 1.

DL utilizes artificial neural networks with multiple layers to learn complex patterns from large datasets, where “deep” refers to the number of layers. On a concept level, a neural network can be considered as a function approximator. Given an input, each layer in a neural network extracts (or learns) some features which then become input to the next layer. There are different types of neural networks. Given that the input in our work is an image of marine magnetic anomalies. We chose convolutional neural network (CNN) because of its proven success in a wide range of compute vision applications. Figure 2 summarizes the different layers in a CNN, using an example of recognizing the object in an image. For a neural network to correctly identify an animal in an image, it relies on convolutional layers to pull out crucial features. These layers function by “sliding” numerous kernels (or filters, like the yellow boxes in Fig. 2) across the input. Each individual kernel is responsible for identifying and highlighting a particular feature. Typically, a series of these convolutional layers is needed to build a comprehensive set of features crucial for making correct predictions. Pooling is often applied after a convolution layer to preserve essential features while minimizing computational cost. After a series of convolutional and pooling layers extract all the relevant features, the resulting feature maps (typically small 2D matrices) are flattened into a single 1D vector. This vector is then fed into several dense layers, also known as fully connected layers (as illustrated in Fig. 2). These dense layers combine the high-level features learned by the convolutional layers to make the final predictions. To introduce nonlinearity into the network, an activation function is applied after both convolutional and dense layers. The most common choice is ReLU (Rectified Linear Unit) or its variant, Leaky ReLU, also depicted in Fig. 2. For classification tasks, the final layer usually employs a sigmoid activation for binary classification problems or softmax for multi-category classification. At the training stage, the weights and biases associated with each kernel (in convolutional layers) and neuron (in dense layers) are updated by minimizing a loss function through gradient-based optimization algorithms, such as Adam^35^.

To train a CNN model, a massive set of labeled images is necessary. Indeed, over one million labeled images from ImageNet were used to train AlexNet^36^, VGG16^37^, ResNet-50^38^ etc. However, in marine magnetics as well as many other Earth science applications, labeled images are typically on the order of hundreds to thousands. Due to the small quantity of training images, the CNN model easily overfits the training data, in which case the trained DL model will not generalize well to new data. To overcome the challenges, deep transfer learning has proven effective when only limited training data is available^39–43^. This is also the strategy that we employed in our work to improve the performance of our CNN model.

### Transfer learning

**Fig. 3:**
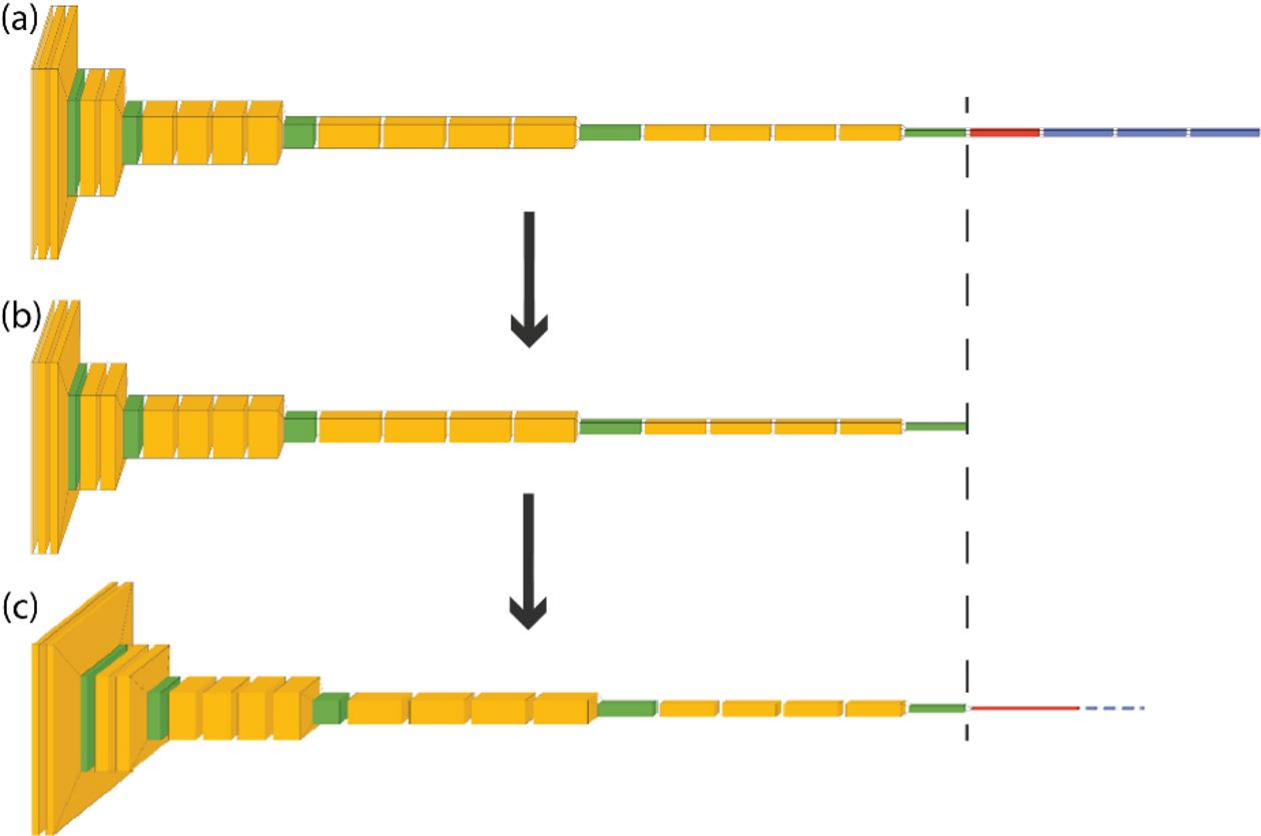
A schematic to illustrate transfer learning using VGG19. (**a**) Architecture of the VGG19 model. Yellow: convolution layers. Green: max pooling layers. Red: flatten layers. Blue: dense (or fully connected) layers. The convolutional layers in VGG19, shown in (**b**), are directly used in the neural network architecture for the target task (**c**).

The fundamental concept of transfer learning involves leveraging convolutional kernels (including their weights and biases) learned from a source task and applying them to a new, target task. In our work, the target task is to classify the marine magnetic anomalies. This methodology relies on a pre-trained model where the learned kernels are directly integrated into the target task architecture. Several prominent pre-trained convolutional networks, trained on the extensive ImageNet dataset, are readily accessible. These include Inception V3, AlexNet, various ResNet architectures (e.g., ResNet18, ResNet34, ResNet50, ResNet101), VGG models (VGG16, VGG19), MobileNet, and MobileNet V2. In Fig. 3, we use VGG19 as an example to illustrate the idea of transfer learning. Figure 3a presents the architecture of the VGG19 model. The yellow blocks represent the convolution layers, green max pooling layers, red flatten layers, and blue dense (or fully connected) layers. The convolutional layers in VGG19, shown in Fig. 3b, including all the learned kernels, are directly used in the neural network architecture for the target task in Fig. 3c. Four dense layers are added with ReLU activations except the last layer where a sigmoid activation is used. The weights and biases associated with these dense layers are learned by minimizing a loss function based on the limited labeled training data set for the target task.

## Workflow

**Fig. 4:**
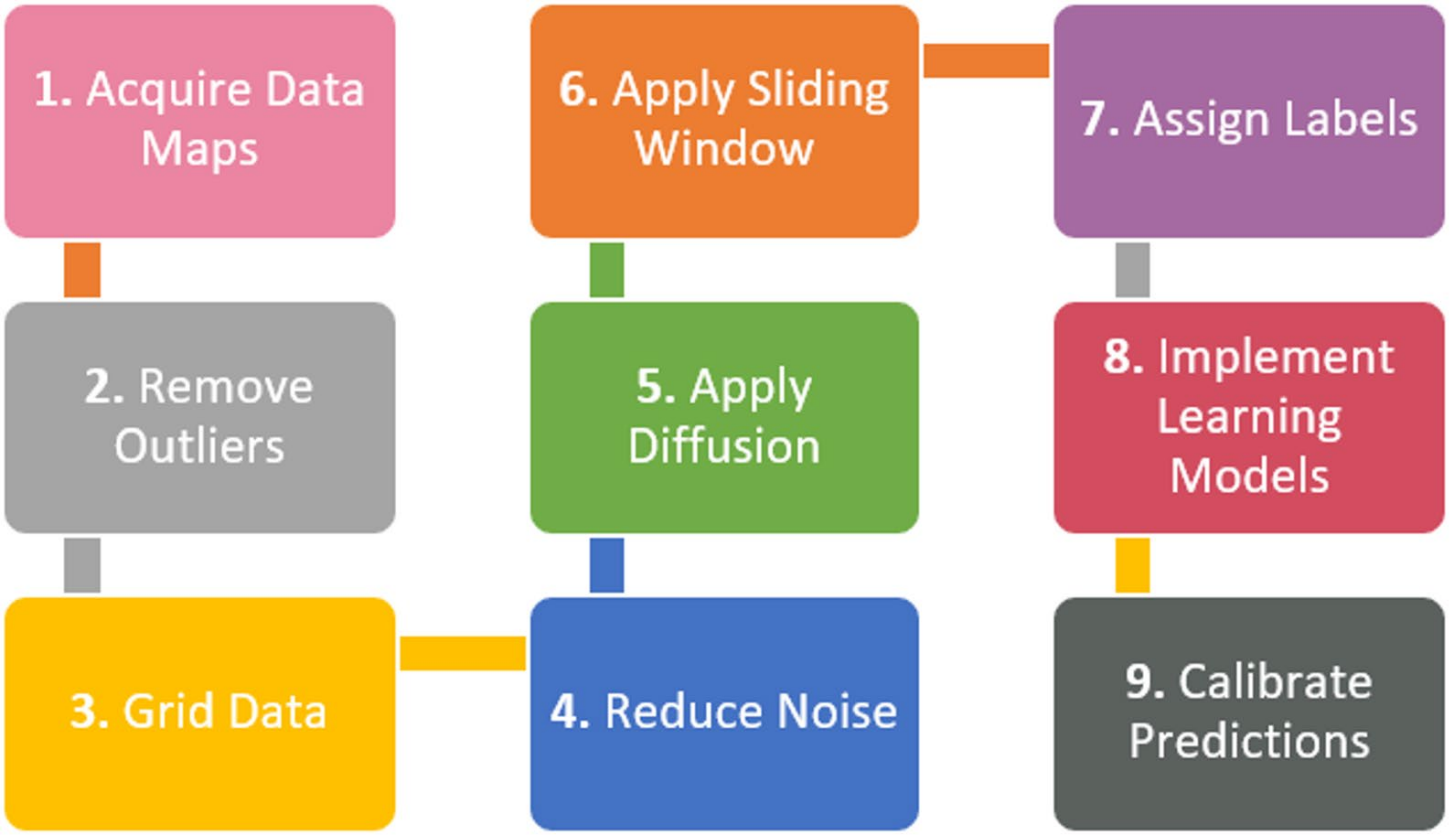
The new workflow for interpreting marine magnetic anomalies. Different from standard workflow that relies on visual analysis, this new workflow features singular value decomposition to reduce noise (Step 4), anisotropic diffusion to enhance continuity of magnetic anomalies (Step 5) and deep transfer learning (Step 8) to identify LMAs even when the size of labeled training data set is small.

Based upon anisotropic diffusion and transfer learning described in the previous section, we proposed a new workflow to interpret marine magnetic anomalies. Figure 4 summarizes the steps in our workflow. Below we explain each step.

### Data preprocessing

**Fig. 5:**
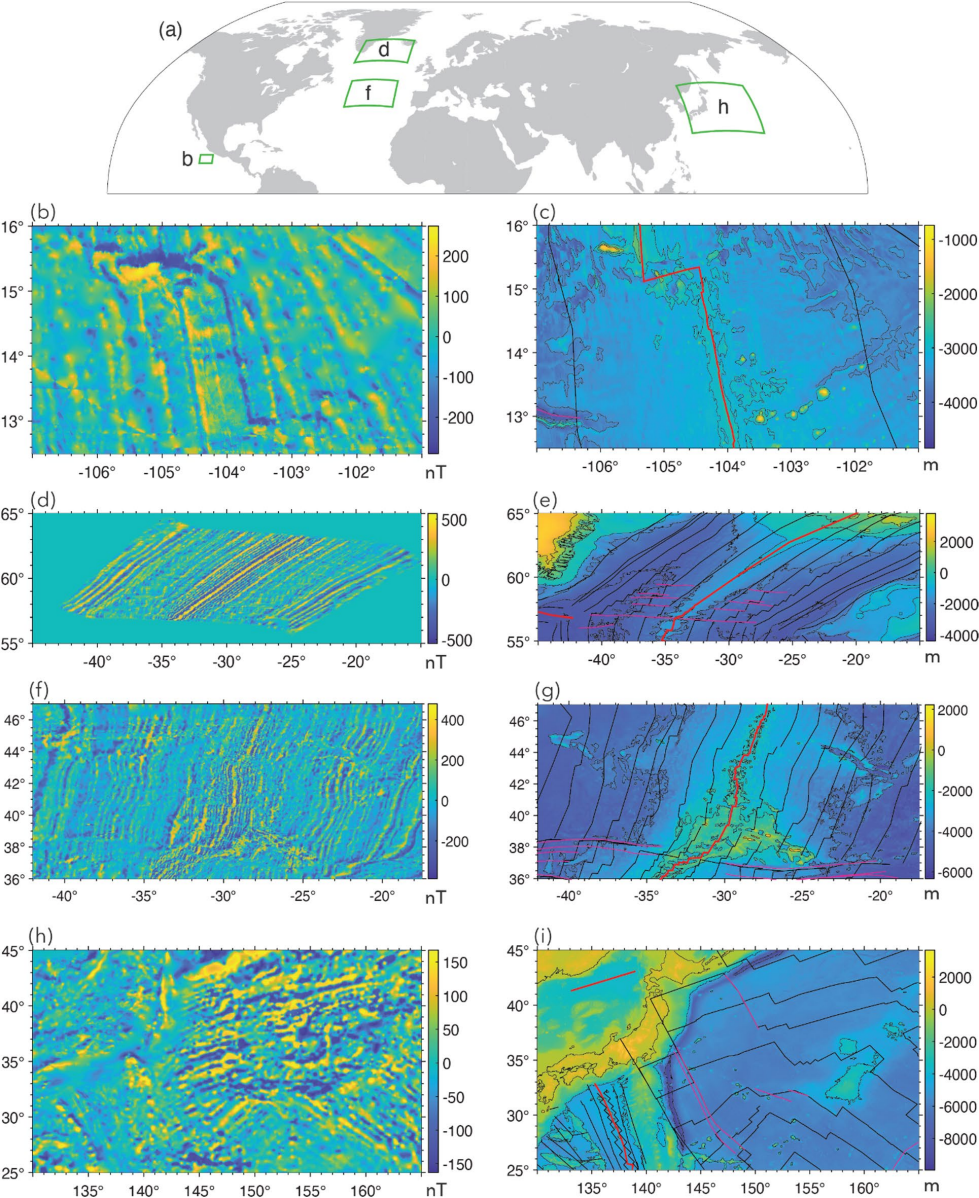
The marine magnetic anomalies from the four study areas and the corresponding bathymetry maps^80^. (**a**) Locations of the four study areas in our work. Marine magnetic anomalies from East Pacific Rise (**b**), Reykjanes Ridge (**d**), Azores Plateau (**f**), and Shatsky Rise (**h**). The corresponding bathymetry maps are placed to the right of each anomaly map. The unit of marine magnetic anomalies is in nanotesla (nT), and the unit of bathymetry measurements is in meter (m). Black lines: magnetic isochrons^81^; Solid red lines: ridges^82^; thin red lines: fracture zones^83^. The plots were generated using MATLAB R2021b^84^ and GMT 6^54^.

**Fig. 6:**
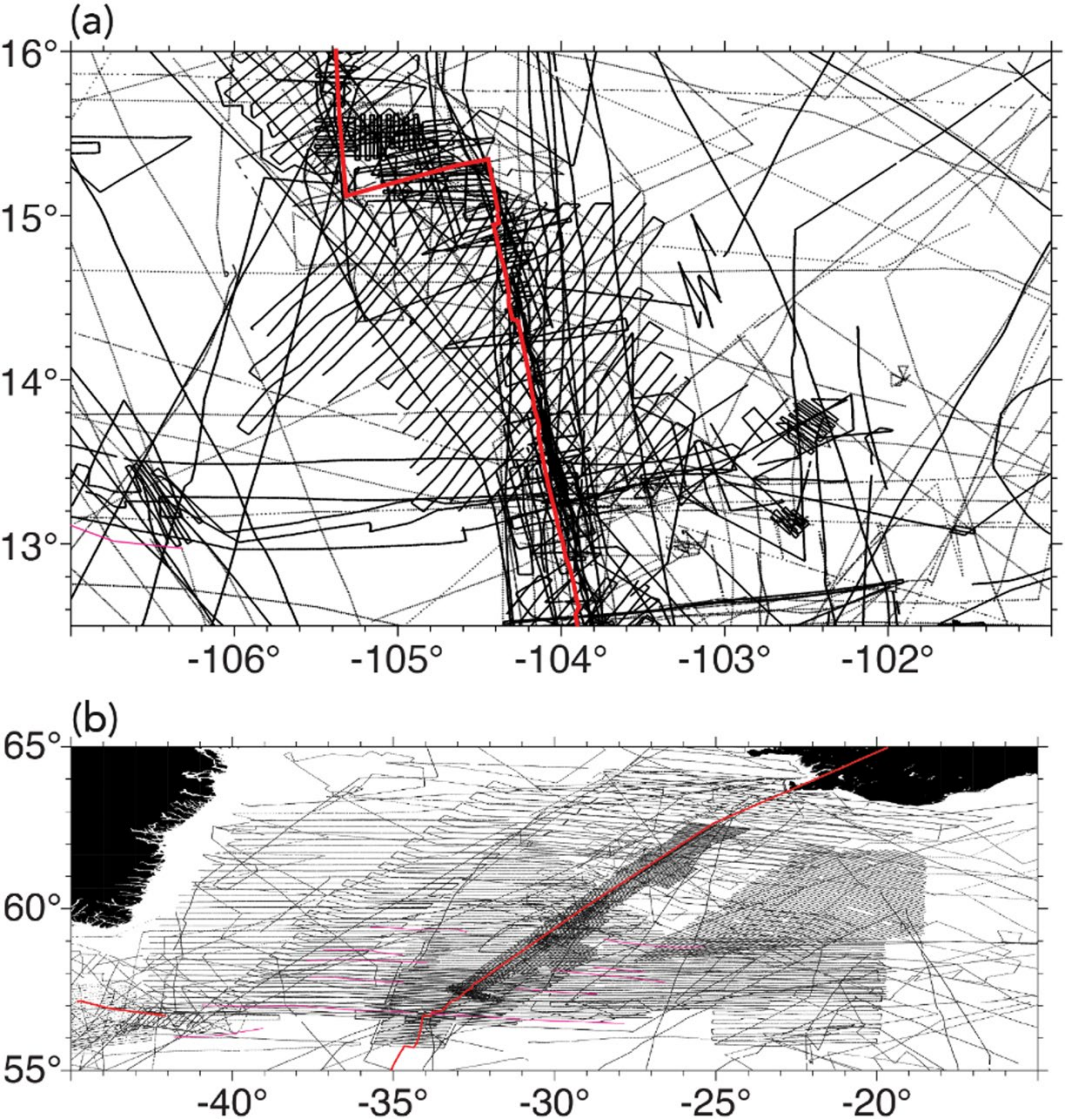
The tracklines for (b) EPR data and (d) Reykjanes data (NCEI marine geophysics data archive). Red lines represent ridges^82^. Thin red lines indicate fracture zones^83^. The plots were generated using GMT 6^54^.

 Steps 1–3 involves data preprocessing. The first step is to obtain marine magnetic anomaly data grids. We selected four study areas (Fig. 5) where LMA are obvious and a well-constrained data grid is available for a large area: (1) East Pacific Rise^44^ (EPR), (2) Reykjanes Ridge^45^, (3) Azores^46^, and (4) Shatsky Rise (EMAG2v3^47^). The first three of these are available as 1-arcmin grids from the cited references (Table S1). The EMAG2v3 data from NCEI (National Centers for Environmental Information) are gridded at 2-arcmin resolution. The magnetic anomalies from EPR (Fig. 5b), are characterized by dominantly linear magnetic anomalies, but it is also obvious that some of the LMAs are disrupted either due to sparse data coverage, measurement errors or strong localized anomalies. For magnetic anomalies from Reykjanes Ridge (Fig. 5d), we observe well-defined LMAs, but linearity is disrupted ~ 4°−5° on either side of the ridge where tectonic reorganization distorts magnetic anomalies^45^. The EPR data shows relatively sparse data coverage away from the ridge (Fig. 6a), whereas Reykjanes data set shows very dense coverage (Fig. 6b). We used the anomalies from EPR and Reykjanes Ridge areas for creating labels and training our deep learning model. The anomalies from the Azores and Shatsky Rise study areas in Fig. 5f and h, which contain LMA but also clear nonlinear features, were used for testing how well our best-performing neural network, trained on magnetic anomalies from EPR and Reykjanes Ridge, generalizes to magnetic anomalies from new areas. Figure 6 presents the tracklines from the EPR and Reykjanes regions. The tracklines for the Azores and Shatsky Rise regions are shown later, in Fi.gs. 10 and 11, where we compare them with the network predictions.

**Fig. 7:**
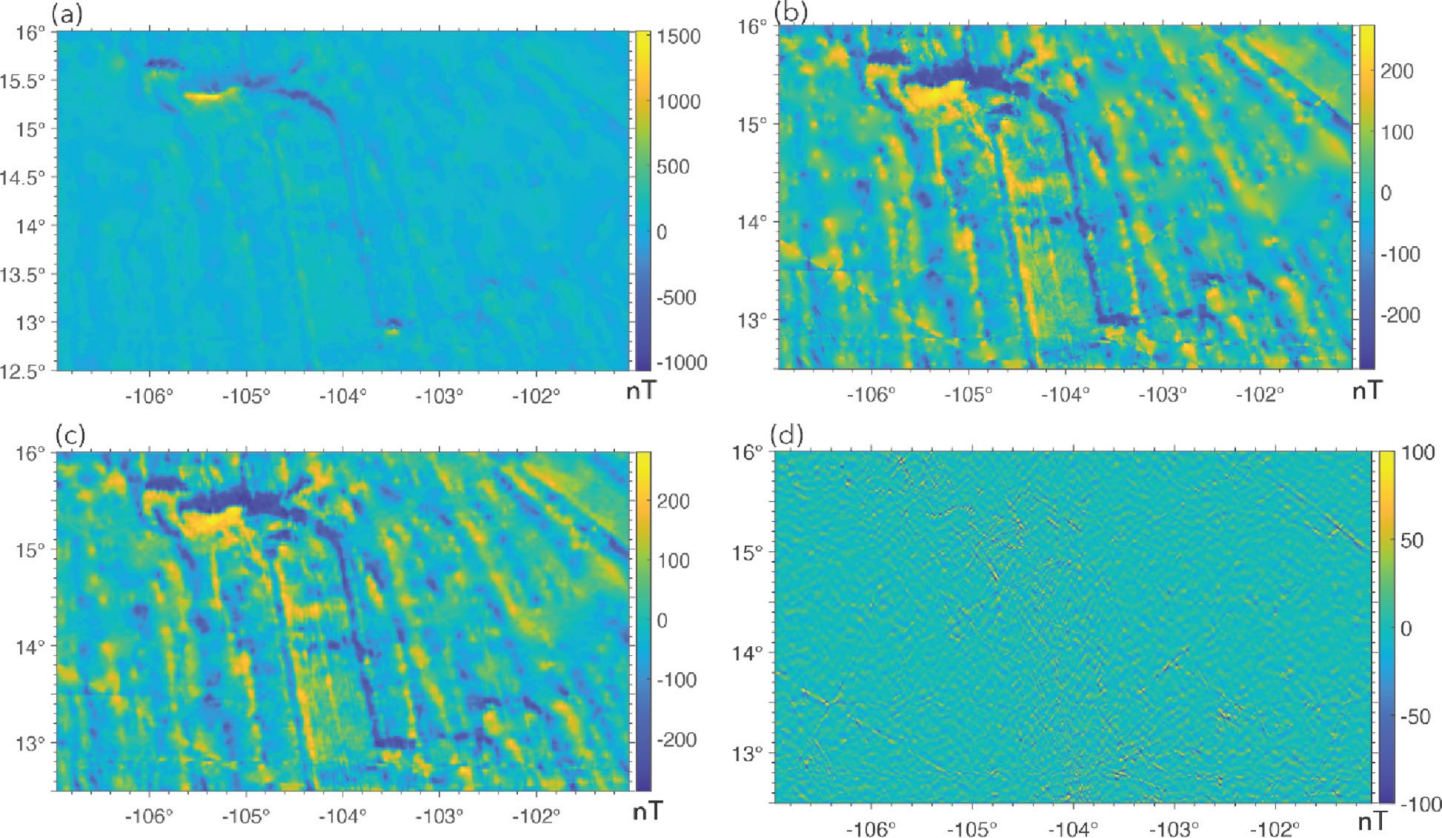
An illustration of data processing procedures in our workflow. (**a**) Original magnetic anomalies at EPR, (**b**) magnetic anomalies after removing outliers and gridding, (**c**) anomalies after removing noise using SVD, (**d**) the difference between anomalies in (**b**) and the de-noised anomalies in (**c**).

In Step 2, we remove outliers in the magnetic anomalies. We consider extremely large (positive) and small (negative) anomaly values as outliers. The existence of outliers significantly widens the dynamic range of anomalies in an area. As shown by Table S1, the dynamic ranges of magnetic anomalies in our study areas are wide. This poses challenges for training, because such wide ranges of anomaly values suppress weaker yet meaningful linear anomalies, as shown by Fig. 7a. It is clear that the existence of very strong anomalies in the upper left region makes the widespread LMAs in the EPR area much less recognizable. The wide dynamic range also creates numerical challenges for anisotropic diffusion (at Step 5). The reason is that these outliers can create extremely sharp, localized gradients, which anisotropic diffusion might interpret as highly significant edges and preserve them, rather than smoothing them out as noise^15,29,48,49^.

 To remove the outliers, we first computed the interquartile range (IQR) which is defined as IQR = Q3-Q1, where Q1 is the first quartile and Q3 the third quartile. Once we obtained IQR, we define outliers as any magnetic anomaly values falling outside of the range [Q1-1.5 IQR, Q3 + 1.5 IQR]. This is a widely accepted method and is commonly referred to as the Tukey’s method or the 1.5 IQR rule in statistics^50–53^. We applied this procedure to the magnetic anomalies from each study area. After the outliers were removed, the dynamic range of magnetic anomaly values at each study area is much narrower, as shown in Table S2.

In Step 3, we replaced the missing values (from Step 2) with interpolated ones. We implemented spline interpolation, also referred to as gridding in Generic Mapping Tools (GMT^54^. One important user-specified parameter for gridding is the grid interval. There are two considerations. First, the grid interval must be small enough to allow for accurate representation of fine-scale magnetic anomalous features. According to the Nyquist-Shannon sampling theorem, the sampling frequency must be at least twice the highest frequency present in the signal. In spatial terms, this means that the grid interval (i.e., sampling rate in the spatial domain) must be no greater than one half of the width of the thinnest magnetic anomalies. Due to the distinct tectonic processes, especially the different spreading rates, the magnetic anomalies in the four study areas exhibit varying scales or widths. While the Reykjanes Ridge and Azores share comparable magnetic anomaly widths of approximately 20–30 km, the fine-scale anomalies at the East Pacific Rise (EPR) are significantly narrower, around 10 km wide. Conversely, Shatsky Rise displays broader anomalies, measuring approximately 40 km in width.

The second consideration is that each window, consisting of 250 by 250 data points (or, pixels), should ideally contain four to six linear anomalies. Too many anomalies within a window will complicate the neural network predictions. On the other hand, having too few (e.g., only one or two) anomalies might result in an excessive zoom-in, thereby losing valuable spatial contextual information critical for correctly differentiating linear from nonlinear anomalies.

Based on the two considerations and the dominant anomaly widths in our study areas, we adopted a grid spacing of 0.24 arcmin for EPR and Reykjanes Ridge, 0.36 arcmin (~ 0.67 km) for Azores, and 0.72 arc-minute (~ 1.33 km) for Shatsky Rise (Table S2). In Fig. 7b, we display the magnetic anomalies at EPR after removing outliers and gridding. It shows that Steps 2 and 3 in our workflow help bring out the LMAs. Visually, they are much easier to recognize, which we believe will also make it easier for neural networks to make correct predictions.

### Noise reduction and anomaly enhancement

 Step 4 in our workflow is to remove noise from the gridded magnetic anomalies which are always contaminated by various sources of noise (e.g., cultural noise from ships, instrument noise, interpolation errors due to uneven and sparse data coverage, etc.). Singular Value Decomposition (SVD) is a powerful matrix factorization technique widely used in signal processing for noise reduction^55,56^. The core idea behind SVD for noise reduction is that dominant and coherent signals tend to be represented by a few large singular values, while random or incoherent noise is associated with smaller singular values. To remove noise, we simply performed SVD on the data matrix consisting of the gridded values and constructed a low-rank approximation by setting the smaller singular values (those below a certain threshold) to zero. The random and incoherent patterns associated with these small singular values are then removed. In our work, we retained the k largest singular values that preserve ~ 98% of the data variance while setting the rest to zero (rank-k approximation). For the EPR data, k = 47, and for the Reykjanes, Azores and Shatsky Rise data, k = 87 (Table S3). This ensures minimal loss of essential information. The resulting 2D output was used as input for anisotropic diffusion in the next step. In Fig. 7c, we display the magnetic anomalies after removing noise using the low-rank approximation method. The difference between the denoised anomalies and the original ones is shown in Fig. 7d.

Compared with other noise reduction methods, the SVD method has two advantages. First, it does not require prior knowledge of the noise statistics or signal characteristics. Secondly, it can reduce white noise, colored noise, and even coherent interference (like cultural noise) if it exhibits different spatial/temporal patterns than the signal. In Earth science, it has been successfully applied to denoise seismic data^57,58^. To the best of our knowledge, it has not been used to denoise marine magnetic measurements before.

The denoised magnetic anomalies are then input into an anisotropic diffusion routine in Step 5. Our implementation of anisotropic diffusion, following Weickert^16^, is guided by 3 parameters – local scale or noise scale (σ), integration scale (ρ), and diffusion time step (T). In the first step of anisotropic diffusion, an initial structure tensor is constructed to describe the local structure and its orientation. To make the estimation of the local structure robust to noise, the image is first convolved (or smoothed) with a Gaussian kernel. The noise scale σ is the standard deviation of the Gaussian kernel. If σ is too small, Gaussian smoothing is very localized, and the estimated structure tensor becomes highly sensitive to noise. If it is too large, it might blur out important fine-scale features in the magnetic anomalies. After computing the initial structure tensor (which is based on local gradients smoothed by σ), the elements of this tensor themselves are further smoothed by convolution with another Gaussian kernel, this time with standard deviation ρ. This ρ is the “integration scale.” Smoothing the structure tensor with ρ helps to aggregate this local information over a larger neighborhood, creating a more stable and robust estimate of the image’s “coherence” or “texture” at a broader scale. The ρ value guarantees stable orientation estimates and should be equal to or larger than the image texture scale, and must not be underestimated^16^. The time step T is simply the number of times diffusion is performed. The larger the T, the smoother the output image. The σ and the ρ must be adapted to the noise and the texture scale of the input image. The ρ reflects the characteristic size of the texture, which is usually large in comparison to the σ. The parameters used for our data maps are listed in Table S3. The choice of ρ, which is smallest for the EPR data and largest for the Shatsky Rise data, reflects the characteristic texture scale (anomaly width). Figure 1c and d show the magnetic anomalies at EPR before and after diffusion.

### Creating labeled training images

**Fig. 8:**
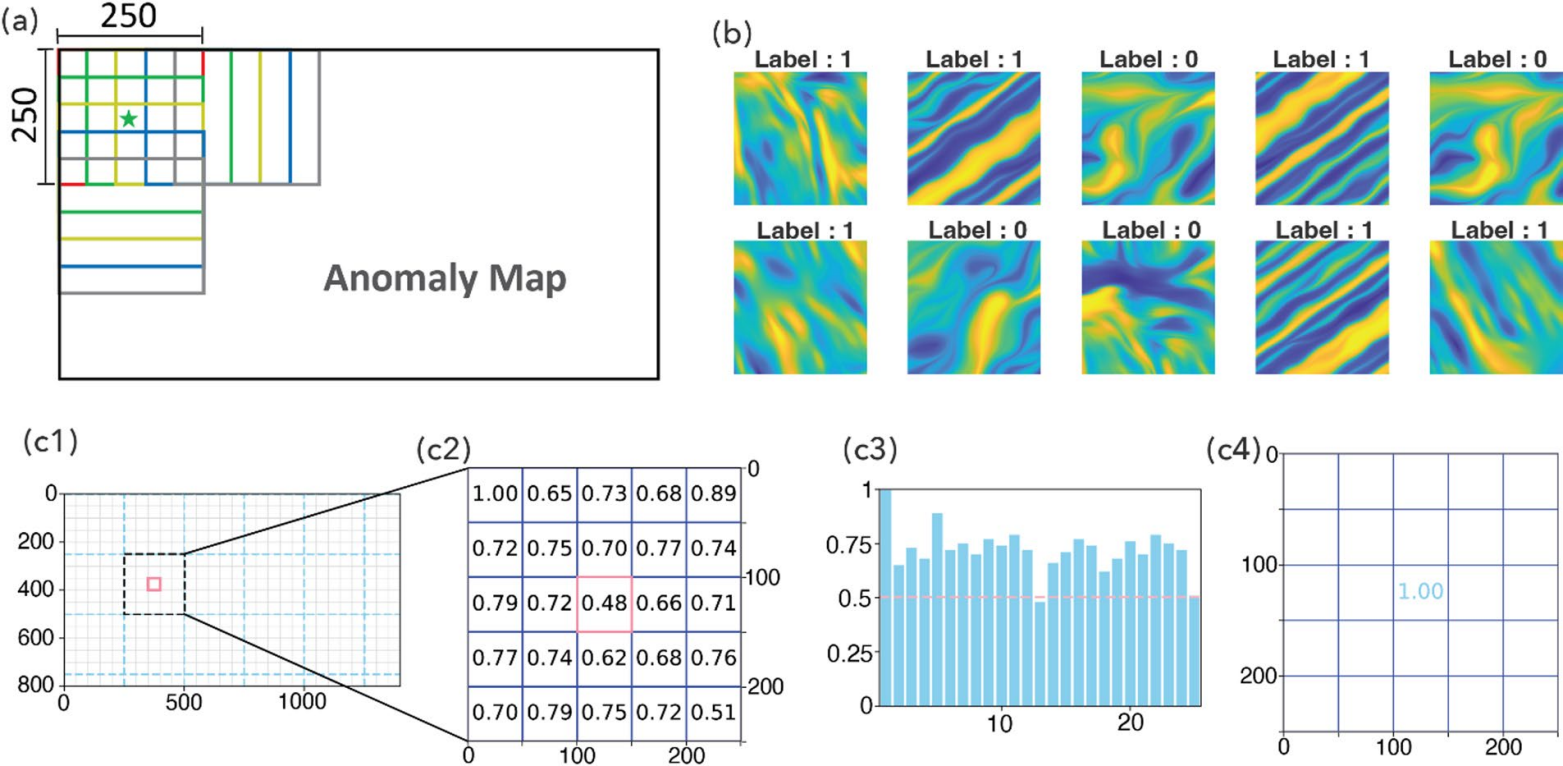
(**a**) Schematic of a 250 × 250 window sliding over an anomaly grid, with a stride of 50 pixels. Colored squares mark 5 horizontally and 5 vertically overlapping windows. The green star marks a 50 × 50 square with 24 neighboring windows. (**b**) Ten examples of 250 × 250 magnetic images with assigned class labels. (**c**) Aggregating and calibrating final predictions: (c1) a 250 × 250 window, (c2) the raw prediction in the center of the 250 × 250 window as well as the predictions from its neighbors, (c3) distribution of all the predictions in the 250 × 250 window. Red dashed line represents a cut-off value of 0.5, (c4) prediction in the center of the 250 × 250 window adjusted to the maximum value in the 250 × 250 window.

After removing random noise and enhancing the continuity of LMAs, in Steps 6–7, we created training images and the associated labels using the resulting anomaly maps at EPR and Reykjanes Ridge in Fig. 5b and c, respectively. In Step 6, we took a moving window approach which divided each data map into many small overlapping windows. The selection of window size is important. An insufficient window size can lead to the amplification of minor nonlinearities, thereby misrepresenting even inherently linear magnetic anomalies as nonlinear. Conversely, an oversized window might encompass too many anomalies—both linear and nonlinear—making accurate classification impossible. In addition, an excessively large window size results in a low spatial resolution in the resulting classification map. A 250 × 250 window size was chosen after visual inspection of both the original and diffused 2D anomaly maps. This dimension was deemed appropriate as it is large enough to adequately capture the spatial patterns of LMAs, yet sufficiently constrained to include a representative sample of anomaly patterns—typically four to six peaks—within each window. In degrees, the 250 × 250 window size translates to 1°×1° for EPR and Reykjanes Ridge, 1.5°×1.5° for Azores, and 3°x3° for Shatsky Rise. To make full use of the anomaly patterns in the EPR and Reykjanes Ridge and to create as many training images as possible, we adopted a stride of 50 pixels when moving the current window to the next location. Figure 8a illustrates a 250 × 250 window sliding over an anomaly grid with a stride of 50.

Step 7 is to assign a label to each training image created from the previous step. Marine magnetic experts in our team visually examined each image, including both the original and the diffused versions, and manually assigned a label to each. A label of ‘1’ was assigned to a 250 × 250 window if at least 70% of its magnetic anomalies exhibit linear patterns. A label of ‘0’ was given if at least 70% were determined to be nonlinear. This 70% cutoff was established to ensure clear and unambiguous classification by human experts. Windows where neither linear nor nonlinear anomalies predominate (based on the 70% criterion) were regarded as ambiguous and excluded from the training dataset. From the 288 windows at EPR, we successfully labeled 277. At Reykjanes Ridge, we labeled 574 out of over 4,000 windows, focusing on those exhibiting clear linear or nonlinear patterns. Therefore, we created 851 labeled marine magnetic images which were used in the next step of training neural networks. In Fig. 8b, we show 10 examples of 250 × 250 magnetic images and their labels.

### Training and evaluating neural networks

In step 8, we randomly split the labeled magnetic images into three sets, namely, training, validation and test sets, in a 6:2:2 ratio. The training set contains 510 labeled images. Validation and test sets each contain about 170 labeled images. Before inputting magnetic images into neural networks, we normalized each image to a range of [0, 1] using the standard min-max normalization method for faster convergence during training. This was done for all images in training, validation and test sets. We point out that, because outliers in our magnetic data have already been removed in Step 2, there is no need to use robust scalers or transformations. Using the training and validation sets described above, we trained three DL models, i.e., a standard CNN, transfer learning without anisotropic diffusion and with it. The predictive performance of the three DL models was evaluated and tested against each other using the test data set. The best performing DL model, i.e., transfer learning based on diffused images, was then applied to magnetic images at Azores and Shatsky Rise. We present and discuss the results in the section Results.

### Aggregating and calibrating final predictions

Once training was complete, in Step 9, we applied our best-performing deep learning model to magnetic images from Azores and Shatsky Rise. We used the same 250 × 250 moving window approach, but with a smaller stride of 10 pixels instead of 50. Here’s how it worked: We started a 250 × 250 window at the top-left corner of each magnetic anomaly map. The neural network then made a prediction (a floating-point number between 0 and 1). We then moved the window 10 pixels to the right (or down once we reached the rightmost location) and made the next prediction. The prediction from each window was assigned to its central location (a 10 × 10 area in the center of each window). If the prediction was greater than 0.7 or smaller than 0.3, we considered the neural network confident and accept its prediction. However, for predictions between 0.3 and 0.7, we aggregate the predictions from its neighboring windows and adjust the final prediction for that location. Because we used a stride of 10 pixels, any 10 × 10 area in the interior of a study area is covered by 25 × 25 = 625 windows, and therefore, is associated with 625 predictions. When adjustment is necessary (i.e., when the prediction from the current window falls between 0.3 and 0.7), we use these 625 surrounding predictions for refinement. Specifically:

- If 50% or more of these 625 predictions are greater than 0.5, the prediction for the current 10 × 10 area is adjusted to the maximum possible value (likely 1.0, indicating strong linearity), as shown in Fig. 8c3 and c4.
- Similarly, if 50% or more of these 625 predictions are less than 0.5, the prediction for the current location is adjusted to the minimum possible value (likely 0.0, indicating strong nonlinearity).

In Fig. 8c1-c4, we illustrate the aggregation and calibration procedure. We note that, for the purpose of clarity, we use a stride of 50 pixels in the illustration, instead of 10 as described above in the text.

## Results

### Training

For all the training in our work, we used binary cross-entropy as the loss function and the Adam optimizer^35^ to minimize it. We used the default learning rate of 0.001. The batch size was 32. Each network described below was trained for 25 epochs.

**Fig. 9:**
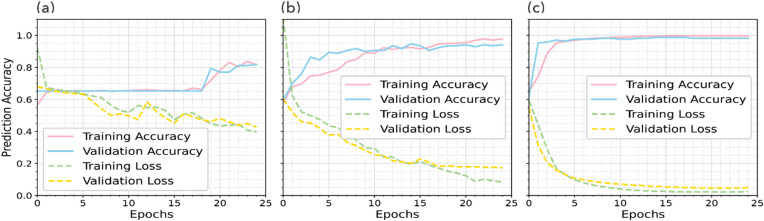
Prediction accuracies and convergence curves for the three different DL models: (**a**) CNN model, (**b**) transfer learning using undiffused anomaly maps, (**c**) transfer learning model using diffused anomaly maps.

We first trained a CNN model which consists of four 3 × 3 convolutional layers, two 2 × 2 max-pooling layers, three dropout layers, four dense layers, followed by a final output layer (Fig. S1a), to predict two probability values (0–1), one for each class. The network has 24.69 × 10^6^ trainable parameters. We used undiffused magnetic anomaly maps in our training data set as the input. The trained CNN model achieved an accuracy of ~ 88% for the training set, and ~ 84% for the validation set (Fig. 9a). The prediction accuracy on the test set was ~ 78%. We think that overfitting might have happened, which is not surprising given the very small size of our training data set.

 Next, we trained a transfer learning model based on VGG19 using the training, validation and test images created from undiffused magnetic maps from EPR and Reykjanes Ridge. It consists of 29 layers (Fig. S1b) including three dropout layers^59^, resulting in a total of ~ 2.52 × 10^6^ trainable parameters. Using TPU provided by Google Colab, it took ~ 7 min to train for 25 epochs. The prediction accuracies are ~ 96%, ~ 95%, and 91%, for training, validation, and test sets, respectively (Fig. 9b; Table S4). They are systematically higher than those from a standard CNN model.

Lastly, we trained another transfer learning model using diffused images. The network consists of 26 layers (Fig. S1c) and ~ 2.52 × 10^6^ trainable parameters. We removed the dropout layers. The prediction accuracies for training, validation and test data sets are ~ 99.9%, ~ 98.8%, and 98.6% respectively (Fig. 9c, Table S4), which are higher than predictions when undiffused images were used in transfer learning. More importantly, the difference between training and test accuracy keeps decreasing from the first to the third DL model. Therefore, transfer learning based on diffused images not only improves the prediction accuracy but also has a somewhat surprising effect of minimizing overfitting, even without dropout layers. We note that the reported prediction accuracy of ~ 99% applies only to test images we generated from magnetic anomalies in the EPR and Reykjanes study areas. In these regions, the network’s predictions matched human interpretations about 99% of the time. However, when applied to areas with more complex tectonic settings, we expect lower accuracy. The key takeaway from the improvement in test accuracy (from 78% to 98%) is that combining transfer learning with anisotropic diffusion substantially enhances the network’s predictive performance.

 In addition to VGG19, we also implemented transfer learning using 9 widely used pre-trained DL models, namely, VGG16, Inception V3, MobileNet, MobileNet V2, AlexNet, ResNet18, ResNet34, ResNet50, ResNet101. For fair comparison, we used diffused images for all the implementations. Table S5 summarizes the prediction accuracies for the 10 different transfer learning models. We observed that VGG19, VGG16, Inception V3, MobileNet and MobileNet V2 exhibited almost same performance. We chose VGG19 as our pre-trained model for classifying marine magnetic anomalies from Shatsky Rise and Azores Plateau. However, it is important to note that the choice of pre-trained models is not unique, as VGG16, Inception V3, MobileNet and MobileNet V2 would have yielded similar results.

### Prediction results for the Azores study area

**Fig. 10:**
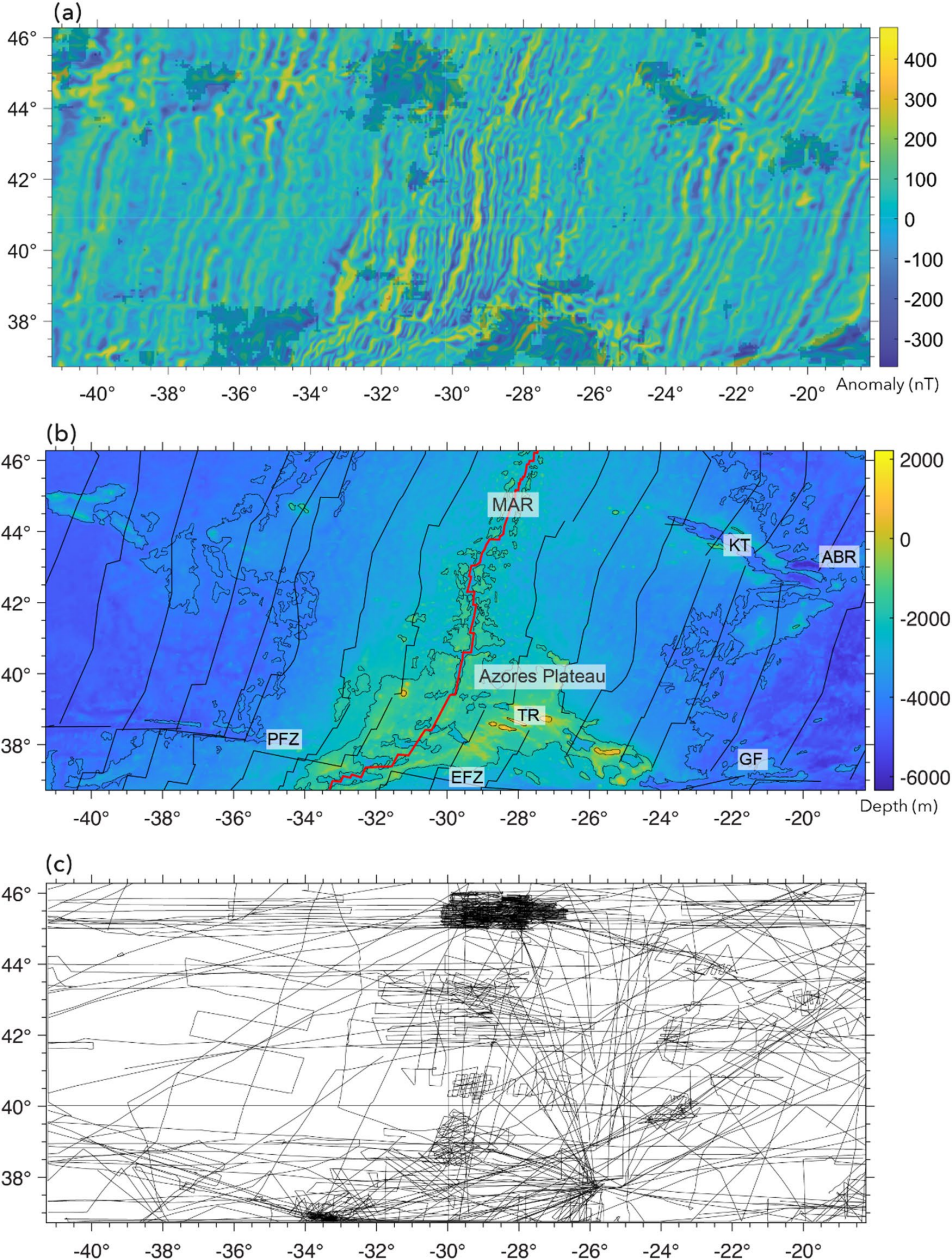
(**a**) Predictions for magnetic anomaly in the Azores study area. Plot shows magnetic anomalies^46^. Areas with dark shade indicate the nonlinear predictions. (**b**) Bathymetric map of the Azores region showing the − 2000 m and − 1000 m contours derived from the SRTM15 + dataset^80^. Magnetic isochrons^81^ are indicated by solid black lines. Annotated features include MAR: Mid-Atlantic Ridge^82^ (solid red line); PFZ: Pico Fracture Zone; EFZ: East Azores Fracture Zone; TR: Terceira Rift; GF: Gloria Fault; KT: Kings Trough; ABR: Azores–Biscay Rise. Azores plateau is mostly encompassed by the − 3000 m contour line. (**c**) Ship tracklines with magnetic data for the study area (from NCEI marine geophysics archive). The plots were generated using MATLAB R2021b^84^ and GMT 6^54^.

The predictions for the Azores study area (Fig. 10a) are consistent with visual inspection. The anomaly map is almost entirely dominated by LMA, except for several nonlinear zones that mark oblique ridges and troughs that cut across and, at places, offset the lineation trends. Nearly 86% of the magnetic anomalies in the Azores region were predicted as linear (Fig. 10a) – these lineations mostly follow the trend (SSW-NNE) of the Mid-Atlantic ridge (MAR). A few nonlinear windows were predicted along two nearly E-W trends across the MAR, in ranges of 36.8^o^- 38.2^o^ N and 43^o^ – 46^o^ N (Fig. 10b). Nonlinear predictions at 46°N, 40.5°W are an artefact of sparse data. Similarly, those at (45°N, 37°W), (45°N, 31°W) and (37°N, 39°W) appear to be artefacts caused by gaps in data coverage. These nonlinear areas do not correlate with any geological or geophysical anomalies. The nonlinear prediction at 42°N, 31°W is an anomaly disruption due to a bathymetric high A zone centered at 37.5°N, 35.5°W, an approximately 200 × 300 sq. km area, displays unclear anomalies and is predicted as nonlinear. Nonlinear predictions at the Azores triple-junction (36.8^o^−39.2^o^ N, 26^o^−30^o^ W) are noteworthy as these zones contain the Azores Plateau – a zone of anomalously shallow bathymetry, split by the MAR in the west, and bounded by East Azores Fracture Zone in the south, and the WNW-ESE trending Terceira Rift to the NE^60,61^ (Fig. 10b). This is a complex, highly tectonized, distributed deformation zone^62^ unlikely to produce LMA. The other notable nonlinear predictions are W-E trending anomalies over the Gloria fault (GF, Fig. 10b), and V-shaped Kings Trough Azores–Biscay Rise (KT-ABR)^63^ (Fig. 10b). These are tectonic features that disrupt magnetic anomalies at places. Anomaly disruptions due to the GF are not continuous and thus prediction is partly nonlinear and partly linear.

### Prediction results for the Shatsky rise study area

**Fig. 11:**
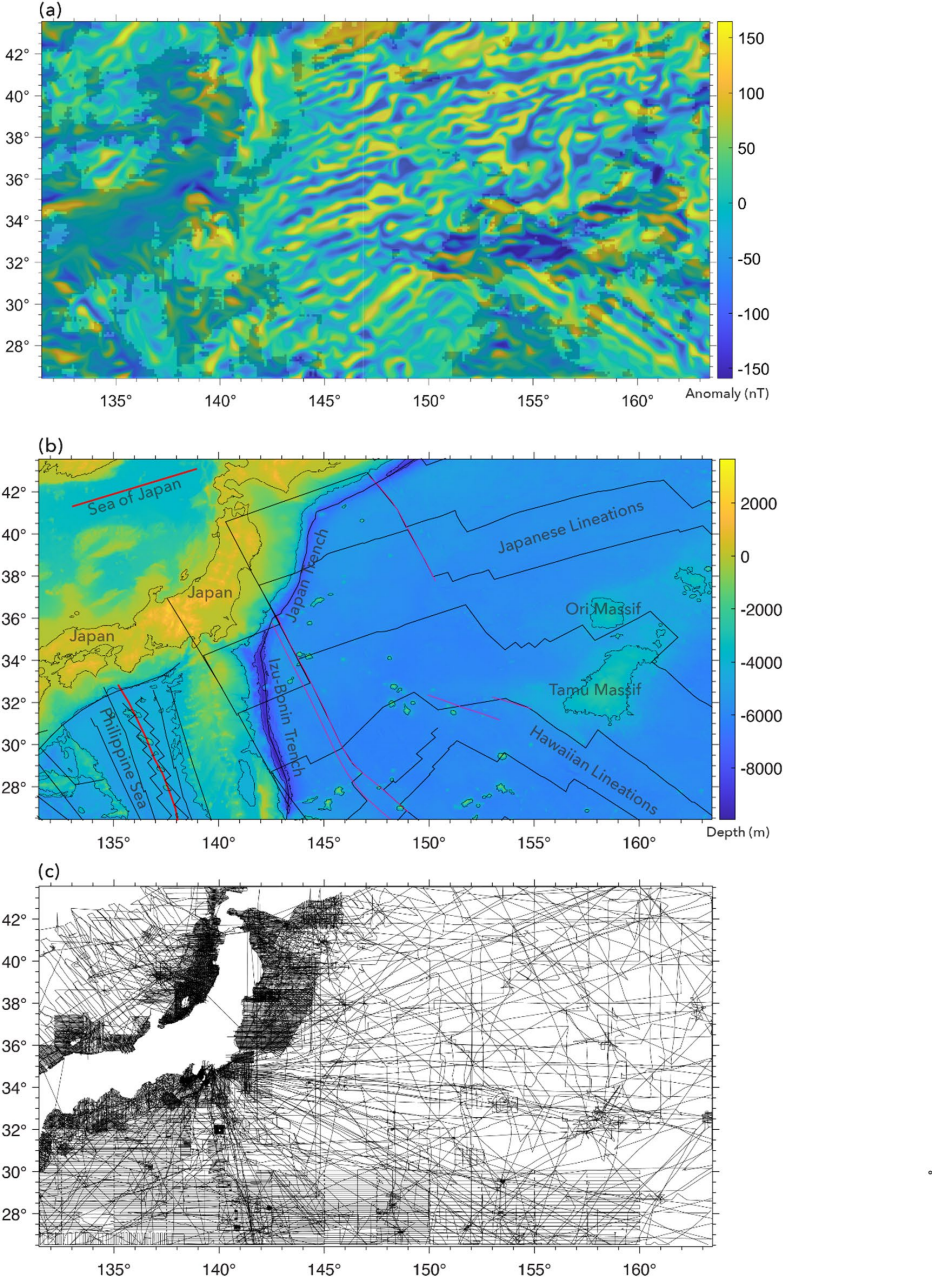
(**a**) Magnetic anomalies and linear/nonlinear predictions for the Shatsky Rise study area. Areas with dark shade indicate the nonlinear predictions; areas with no shade are predicted linear. (**b**) Bathymetric map of the Shatsky Rise shows only the − 4000 m contour derived from the SRTM15 + dataset^80^. Coastline data to the west are taken from the GMT database^54^. Also shown in the map are isochrons^81^ (solid black lines), ridges^82^ (solid red lines), and fracture zones^83^ (thin pink lines). (**c**) Magnetic data tracklines for the region (from NCEI marine geophysics archive). The plots were generated using MATLAB R2021b^84^ and GMT 6^54^.

About 73% of the Shatsky Rise data map east of the trenches is predicted as linear (Fig. 11a) – mostly dominated by the WSW-ENE trending “Japanese” lineations and the NW-SE trending “Hawaiian” lineations^64^ (see Fig. 11b). Linearity is more pronounced in areas of dense data-coverage, and less so in areas where data are sparse and anomaly trends are poorly defined. The LMA prediction around Shatsky Rise is consistent with recent observations that the region is predominantly characterized by LMA^6,65^. Irregular zones characterized by nonlinear behavior occur to the west, east, and south of Shatsky Rise. The eastern nonlinear zone is mainly a result of sparse magnetic data in an area of short anomaly segments, resulting in poor anomaly definition in the EMAG2V3 grid. The zone to the west of Shatsky Rise at 32.7°N, 154°E is an area of contorted magnetic anomalies that may have resulted from tectonic reorganization^65^ and therefore these anomalies may not be linear. To the south of Shatsky Rise, seafloor older than magnetic anomaly M29 is a zone of low-amplitude anomalies that are difficult to correlate^66^. Over Shatsky Rise itself, there is a tectonically complex intersection of the Japanese and Hawaiian LMA. Thus, in some spots over the plateau, the anomalies appear nonlinear.

Nevertheless, the eastern two thirds of the map (Fig. 11a), including Shatsky Rise, is mostly predicted as linear, whereas the western third is dominantly nonlinear owing to the inclusion of western Pacific island arcs, back arc basins, and land (Fig. 11b). There are several areas predicted as linear: the Sea of Japan (NW corner of map) and the basin north of Philippine Sea (SW corner of map). These areas are known to contain LMA from back arc spreading^67–69^ (Fig. 11b).

## Discussion

### User-defined parameters for calibrated predictions

In Sect. 3.5, we explained a calibration procedure for those intermediate predictions falling in the range of [0.3, 0.7]. This procedure involves some user-defined parameters. For example, we used 50% and 0.5 as the cutoff values for adjusting “raw” predicted values from our trained neural network. We have found that they are not always the best optimal values to use. Depending upon the complexity of the magnetic anomalies, some areas might not need any calibration. One such example is in the Shatsky Rise study area. Across the Japan and Izu-Bonin Trenches, the spatial patterns of the anomalies change dramatically. When applied to locations at or near the trenches, the calibration procedure, which simply aggregates predictions from all surrounding windows, would allow the predictions from one side of the trench to unfairly influence the predictions at the other side, resulting in predictions inconsistent with human interpretations. For this reason, we did not apply any calibration to anomalies to the west of the trenches in Fig. 11a. We emphasize that one should not blindly apply the calibration procedure, especially when magnetic anomaly patterns vary significantly in an area of study.

We used 25 × 25 = 625 surrounding windows in Sect. 3.5 for aggregation and calibration. To further understand the extent to which the number of neighboring windows used for adjustment affects the final predictions, we also tried 3 × 3, 5 × 5, 11 × 11, 17 × 17, 21 × 21 neighboring windows. The prediction results are shown in Fig. S2 (for Azores) and in Fig. S3 (for Shatsky Rise). We observe that the predictions do vary but overall, they seem to be robust against the choice of number of neighboring windows.

### Data coverage

Our training data (EPR, Reykjanes) represent both sparse and dense data coverage. Whereas the EPR data show relatively sparse track coverage away from the ridge (Fig. 6a), the Reykjanes data set shows very dense coverage (Fig. 6b). For prediction map data, Azores data has higher density coverage east of MAR compared to that in the west (Fig. 10c). Furthermore, for Shatsky Rise, data density east of 155E is very sparse (Fig. 11c). We included the western region of the Shatsky Rise area map (west of the Japan trench) to test our model performance in regions with few LMA. Nonlinear predictions in our work fall into two main categories: true nonlinear anomalies resulting from tectonic or geologic disruptions and false nonlinear predictions due to insufficient data coverage. Tectonic features such as the Gloria Fault in the Azores study area cause offsets or breaks in LMA and therefore nonlinear predictions. In some areas of low data density and dominantly linear anomalies, the model prediction predicts LMA, despite the sparse data (e.g., west of MAR, Fig. 10b and c). This happens because the linearity of the anomalies is sufficiently represented by the sparse data to make a linear prediction. In contrast, low data density conspires with more complex linear anomalies and the prediction is the opposite (e.g., Hawaiian magnetic anomalies southeast of Shatsky Rise). In the area to the west of the trenches in the Shatsky Rise study area, there are regions of very high data density and predictions are largely nonlinear because the geology is complex and the features are indeed nonlinear (Fig. 11).

### Effect of window size

**Fig. 12:**
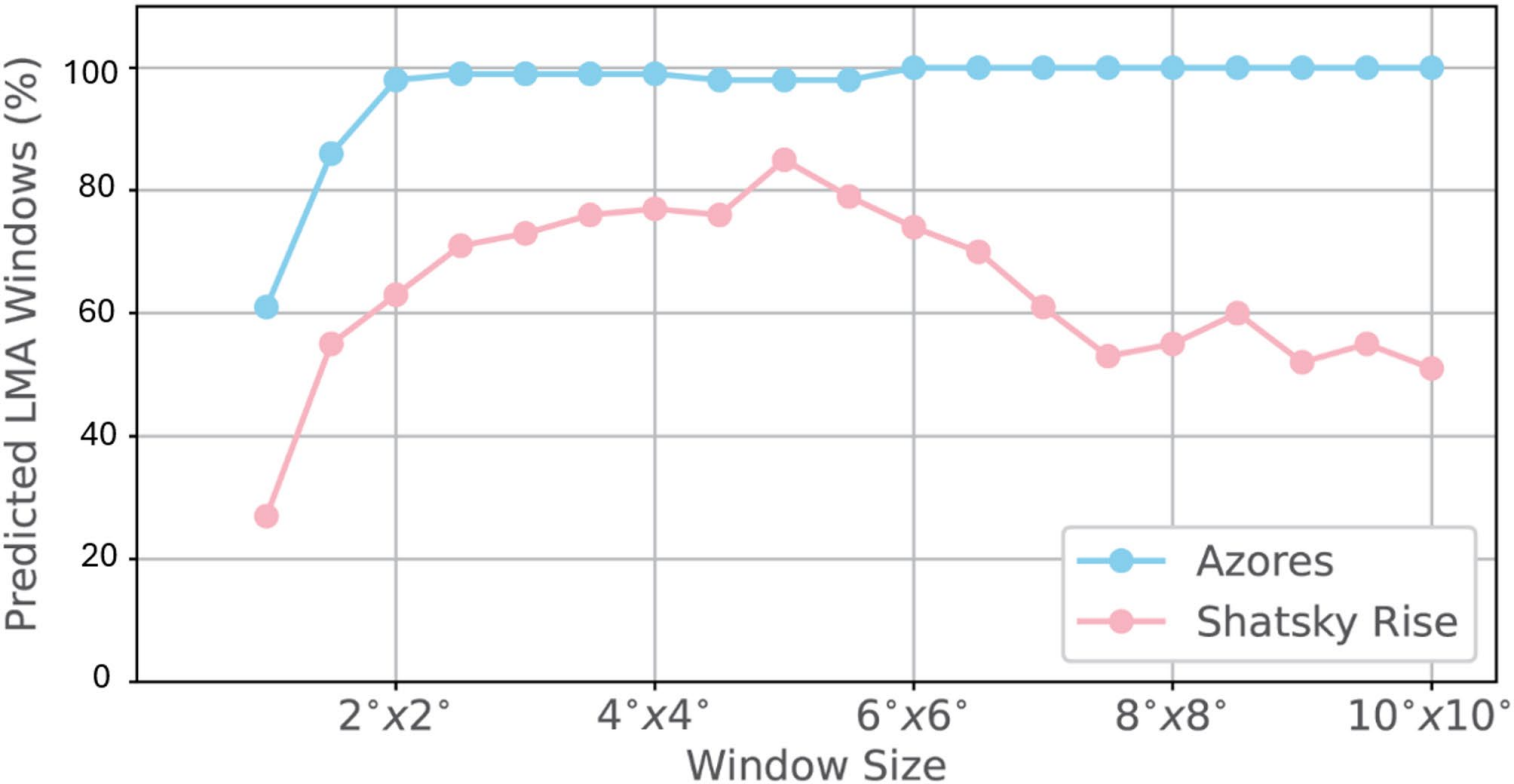
Percentages of predicted LMAs as a function of window sizes for magnetic anomalies at Azores (blue) and Shatsky Rise (red).

The predictions described above are all based on a window size of 250 pixels ×250 pixels. For Shatsky Rise, this translates to 3^o^×3^o^, and for EPR, 1^o^×1^o^. To fully understand the effect of window size on the predictions, we repeated the workflow described in Sect. 4 using several different window sizes. We summarize the results in terms of the percentages of predicted LMAs as a function of window sizes (Fig. 12). For the Shatsky Rise study area, the percentage of predicted LMAs increases with the window size, reaches maximum at 5^o^×5^o^ and shows an overall decreasing trend with further window expansion. When the window size is too small, an excessive zoom-in effect dominates and the spatial context for identifying LMAs is largely missing in the windows, leading to most anomalies being identified as nonlinear. When the window size is too large, it might contain so many anomalies that, unless majority of the anomalies are linear, they will be predicted as nonlinear. This exercise shows that there is a ‘sweet’ zone in which the window size contains the right amount of anomalies for correct predictions to be made.

For Azores study area, we observe a different behavior. The percentage of LMA predictions increases with window size before it plateaus and remains constant at nearly 100%. In this case, the overall linearity of anomalies in the study area allows LMA to overwhelm nonlinear patches. The different behaviors in the Shatsky Rise and Azores study areas are fundamentally because of the complexity of the magnetic anomaly patterns in these two areas. The anomalies at Shatsky Rise are more complex due to the intersection of two distinct sets of lineations, the migrating triple junction in the east and the dominant nonlinear anomalies in the west. Thus, when the window size is large enough, it makes it more likely that some nonlinear anomalies are captured in many windows, causing their predictions to be nonlinear. However, at Azores, the effect of the triple junction, fractures and faults are much more localized, and the anomalies at Azores are predominantly linear or quasi-linear. Even when the window size is very large, each window is still dominated by linear anomalies.

It is important to note that we were not, and should not be, after the maximum percentage of linear predictions when deciding on the optimal window size. Take Azores for example. Had we been interested in maximum LMA predictions, we would have chosen a larger window size such as 4^o^×4^o^. In this case, we would have missed all the nonlinear anomalies caused by the Azores triple junction, the Gloria fault and V-shaped Kings Trough Azores–Biscay Rise.

Unfortunately, there is no automated way of determining the ‘optimal’ window size. We followed a trial-and-error approach, visually comparing the neural network predictions with the known geological and tectonic features in our study areas to make sure that nonlinear anomalies are correctly predicted. One should choose the smallest window size that correctly predicts anomaly character because the method loses spatial resolution as the window expands. Therefore, it is our belief that our deep learning approach should be viewed as a powerful tool to assist, not replace, human experts. After all, we still rely on human experts to create labels for magnetic maps, to decide on ‘optimal’ window size, and to evaluate whether the predictions from neural networks are geologically and tectonically meaningful. We anticipate that this will remain the case for years to come.

### Implications for oceanic plateaus

Oceanic plateaus rise several kilometers above the seafloor and amount to substantial magma transfer from the mantle to the crust^70^. Yet, the formation of Oceanic plateaus is poorly understood primarily due to lack of dense and regular data coverage. Both ridge volcanism^6,71–73^ and mantle plume volcanism^74–78^ have been discussed as likely formation mechanisms. The two eruption styles – ridge volcanism and plume pulse volcanism – should result in differing magnetic anomaly patterns with the former dominated by LMA but the latter potentially more random. Our prediction results confirm the observation that magnetic anomalies over the Shatsky Rise are dominantly linear or quasi-linear^6,65^, suggesting that ridge volcanism similar to seafloor spreading is an important component of the formation of oceanic plateaus. Nevertheless, our analysis also shows areas of nonlinear anomalies resulting from complex tectonics associated with the triple-junction and plate boundary reorganizations associated with its propagation. Our results are consistent with thermodynamic modeling of Zhang^79^, which argues for ridge-plume interaction because we find a combination of linear and nonlinear anomalies.

Model prediction for Azores Plateau area 37°N-40°N, 24°W-34°W is dominantly linear, whereas, regions south of Terceira Rift show some nonlinear predictions as they are in a distributed deformation zone, where LMA fabrics from seafloor spreading are disrupted and distorted by complex tectonics. While both Azores and Shatsky Rise plateaus show dominantly linear predictions, they contain some nonlinear patches as well. The nonlinear predictions that are not due to sparse data coverage appear to be due to tectonic complications. For Azores, it is a distributed deformation zone over a diffuse plate boundary, whereas for Shatsky Rise, it is a rapidly changing spreading ridge system during a time of tectonic reorganization. In both cases, it seems that the plateaus are likely formed by plume-ridge interaction, as the plateaus are thickened crust formed by greater-than-normal ridge volcanism.

## Conclusions

Marine magnetics is fundamental to understanding the formation and evolution of oceanic crust. Despite the widespread success of machine learning across most Earth science subdisciplines, marine magnetics remains one of the few areas where notable progress has yet to be achieved. There are two main challenges when interpreting marine magnetic anomalies using machine learning. First, uneven and sparse data coverage often leads to discontinuities or gaps in otherwise linear magnetic anomalies. Secondly, the marine magnetic anomalies with labels are very limited in quantity. To address these challenges, we have developed a novel workflow that departs from traditional visual analysis. This workflow has two key components:

1. **Anisotropic diffusion**: We employ this technique to enhance the continuity of linear magnetic anomalies, directly addressing the challenge of data discontinuities.
2. **Deep transfer learning**: This approach enables effective predictions even with a limited training dataset, mitigating the issue of small labeled datasets and the associated problem of overfitting. Our results demonstrate that deep transfer learning yields robust predictions even with only 851 labeled training images. Furthermore, using diffused images as input significantly boosts prediction accuracy.

We successfully applied our best-performing model to marine magnetic anomalies at two oceanic plateaus, Shatsky Rise and Azores. Our predictions predominantly show linear patterns, which aligns well with human visual analysis. Importantly, our deep learning model also correctly identifies nonlinear anomalies caused by triple junctions, fractures, faults and island arcs.

This new deep learning approach holds significant potential for uncovering deeper insights into the processes governing oceanic crust formation. Despite these promising results, we underscore that the visual analysis and expert evaluation by human geophysicists remain indispensable to the success and interpretation of our findings.

## Supplementary Information

Below is the link to the electronic supplementary material.

Supplementary Material 1

## Data Availability

The magnetic measurements at East Pacific Rise used in our work are publicly available from Marine Geoscience Data System at [https://www.marine-geo.org/tools/search/Files.php? data_set_uid=24141](https:/www.marine-geo.org/tools/search/Files.php? data_set_uid=24141). The compiled magnetic data at Reykjanes Ridge Reykjanes Ridge are available at Martinez, F. (2023) [https://doi.org/10.5281/zenodo.8072317](https:/doi.org/10.5281/zenodo.8072317). The marine magnetic measurements at Shatsky Rise are available from EMAG2_v3 at [https://www.ncei.noaa.gov/access/metadata/landing-page/bin/iso? id=gov.noaa.ngdc.mgg.geophysical_models: EMAG2_V3](https:/www.ncei.noaa.gov/access/metadata/landing-page/bin/iso? id=gov.noaa.ngdc.mgg.geophysical_models: EMAG2_V3). The Azores data can be obtained from the corresponding author upon reasonable request.
