# Supplementary material for: Enhancing marine magnetic anomaly interpretation with anisotropic diffusion and deep transfer learning
Source: Sci Rep. 2025 Dec 5;16:1185. doi: 10.1038/s41598-025-30926-1 (PMC12789140; doi:10.1038/s41598-025-30926-1)
Supplement: Supplementary file 1 — Supplementary Material 1 [file 41598_2025_30926_MOESM1_ESM.docx]

**Supplementary Information for**

**Enhancing Marine Magnetic Anomaly Interpretation with Anisotropic Diffusion and Deep Transfer Learning**

**J. Ghosh^1^, S. Thoram^1^, J. Sun^1*^, W. W. Sager^1^,**

^1^University of Houston, Houston, TX.

*Corresponding author: Jiajia Sun, [jsun29@central.uh.edu](mailto:jsun29@central.uh.edu)

**Table S1:** Summary of marine magnetic anomalies for the four study areas.

| **Study areas** | **Longitude Range**  **(degrees)** | **Latitude Range**  **(degrees)** | **Anomaly Range**  **(nT)** | **Grid Spacing**  **(arcmin)** | **Spreading Rate (cm/yr)** |
| --- | --- | --- | --- | --- | --- |
| 1. East Pacific Rise | -107, -101 | 12.5, 16 | -1449.1, 1474.6 | 1 | 9.0-10.0 |
| 2. Reykjanes Ridge | -45, -15 | 55, 65 | -1927.9, 2870.4 | 1 | 2.0 |
| 3. Azores | -42, -17.5 | 36, 47 | -2027.4, 1833.7 | 1 | 2.0-2.2 |
| 4. Shatsky Rise | 130, 165 | 25, 45 | -571.6, 527.6 | 2 | 4.0-5.6 |

**Table S2:** Gridding parameters for the anomaly maps. The IQRs, for all four anomaly maps, and the corresponding (GMT-interpolated) resulting anomaly bounds.

| **Study areas** | **Longitude Range**  **(degrees)** | **Latitude Range**  **(degrees)** | **Grid Spacing**  **(min)** | **Anomaly Range (nT)** | | |
| --- | --- | --- | --- | --- | --- | --- |
|  |  |  |  | **Initial** | **After outlier removal** | **Interpolated** |
| 1. East Pacific Rise | -107, -101 | 12.5, 16 | 0.24 | -1449.1, 1474.6 | -241.4, 209.8 | -290, 273.5 |
| 2. Reykjanes Ridge | -45, -15 | 55, 65 | 0.24 | -1927.9, 2870.4 | -444.1, 447.5 | -529.6, 546.6 |
| 3. Azores | -42, -17.5 | 36, 47 | 0.36 | -2027.4, 1833.7 | -311, 297 | -368.7, 389.9 |
| 4. Shatsky Rise | 130, 165 | 25, 45 | 0.72 | -571.6, 527.6 | -160.4, 160 | -162.9, 166.8 |

**Table S3:** Some parameters used for denoising and diffusing magnetic anomalies. σ: diffusion kernel, ρ: diffusion width, T: diffusion time steps.

| **Anomaly Maps** | **Grid Spacing (min)** | **Window Size (degree)** | **Diffusion Parameters** | | | **SVD Rank** |
| --- | --- | --- | --- | --- | --- | --- |
|  |  |  | Sigma (σ) | Rho (ρ) | Time-step (T) |  |
| East Pacific Rise | 0.24 | 1x1 | 1.2 | 2 | 20 | 47 |
| Reykjanes Ridge | 0.24 | 1x1 | 1.2 | 5 | 50 | 87 |
| Azores | 0.36 | 1.5x1.5 | 1.2 | 5 | 100 | 87 |
| Shatsky Rise | 0.6 | 3x3 | 1.2 | 10 | 100 | 87 |

**Table S4:** Prediction accuracies for the three deep learning models.

| **Learning Model** | **Training**  **Accuracy** | **Validation**  **Accuracy** | **Test**  **Accuracy** |
| --- | --- | --- | --- |
| ***CNN*** | 0.8824 | 0.8412 | 0.7824 |
| ***VGG19 based on undiffused images*** | 0.9588 | 0.9471 | 0.9059 |
| ***VGG19 based on diffused images*** | 0.9990 | 0.9882 | 0.9883 |

**Table S5:** Training, validation, and test accuracy for 10 different transfer learning models. Diffused images were used for training, validation and testing.

| **Pre-trained Model** | **Training Accuracy** | **Validation Accuracy** | **Test Accuracy** |
| --- | --- | --- | --- |
|  |  |  |  |
| VGG19 | 0.9990 | 0.9882 | 0.9883 |
| VGG16 | 0.9980 | 0.9882 | 0.9942 |
| Inception V3 | 0.9941 | 0.9824 | 0.9825 |
| MobileNet | 1.0000 | 0.9824 | 0.9865 |
| MobileNet V2 | 0.9995 | 0.9951 | 0.9811 |
| AlexNet | 0.9862 | 0.9824 | 0.9649 |
| ResNet18 | 0.9000 | 0.9235 | 0.9181 |
| ResNet34 | 0.9137 | 0.9118 | 0.9087 |
| ResNet50 | 0.7059 | 0.8176 | 0.8480 |
| ResNet101 | 0.9196 | 0.9294 | 0.9123 |


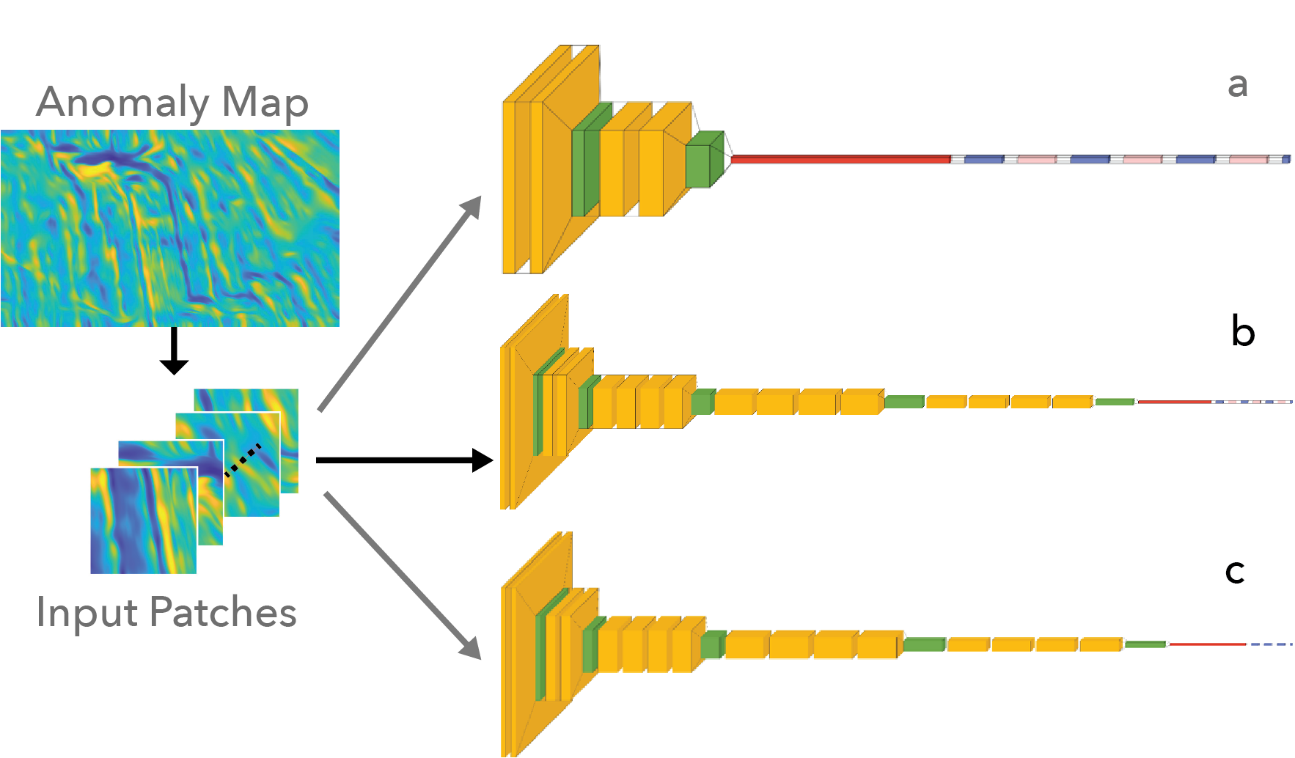


**Figure S1:** Network architectures for a) CNN model, b) deep transfer learning without diffusion, c) deep transfer learning with diffusion. **Yellow**: Convolution layers; **Green:** Max-pool layers; **Red**: Flatten layer; **Blue**: Dense (or fully-connected) layers; **Pink**: Dropout layers.


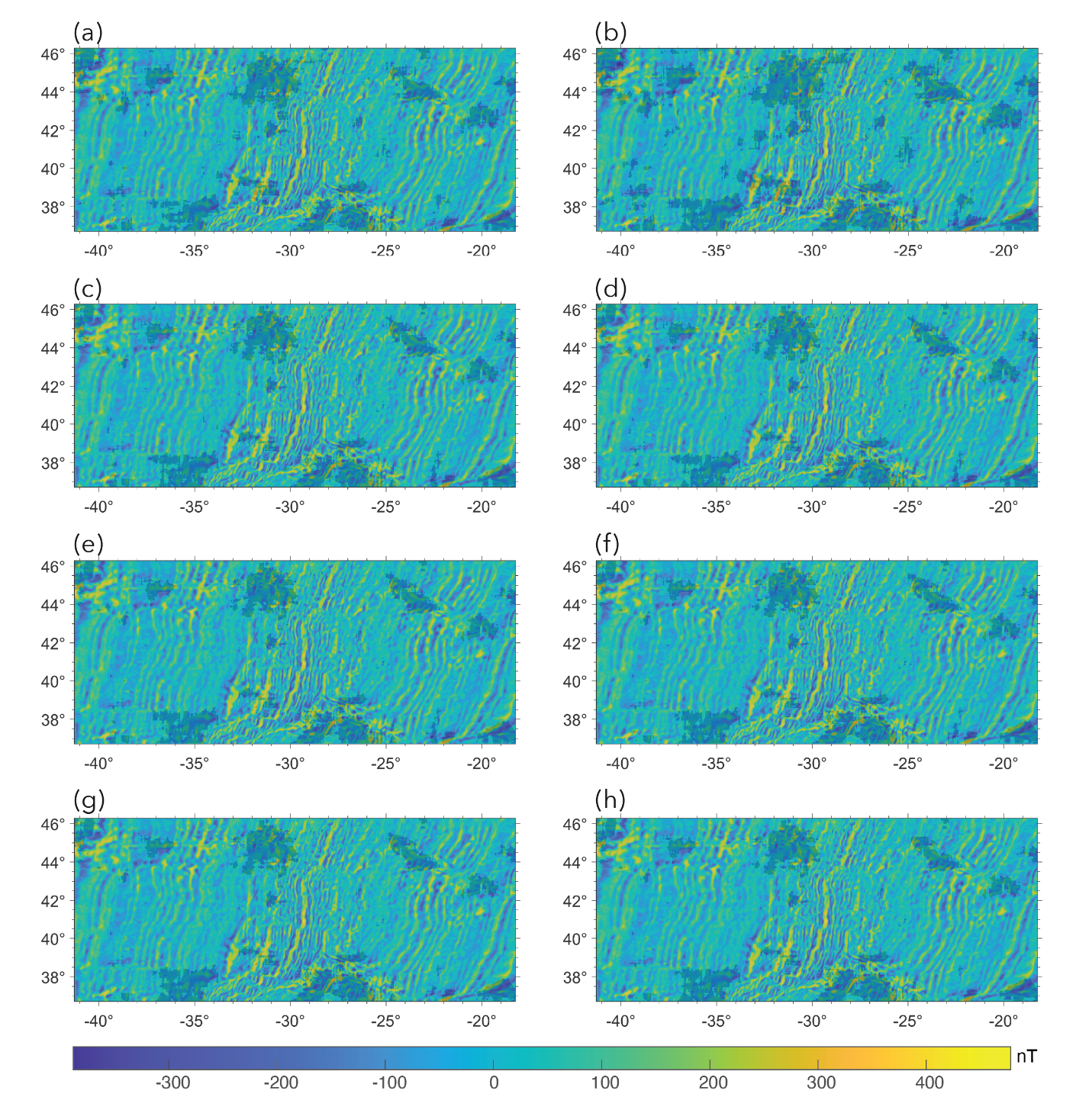


**Figure S2:** Prediction maps for magnetic anomalies at Azores when different cutoff values and various sets of neighboring windows were used. (a) Raw predictions when a cutoff value of 0.5 is used (i.e, any predictions greater than or equal to 0.5 are consider linear, and any predictions below 0.5 are nonlinear). No aggregation and calibration are applied. LMA = 86.80%. (b) Raw predictions when a cutoff value of 0.7 is used. No aggregation and calibration are applied. LMA = 80.23%. (c) Adjusted predictions using 3×3 neighboring windows for aggregation and calibration. LMA = 88.25%. (d) Adjusted predictions using 5×5 neighboring windows for aggregation and calibration. LMA = 88.51%. (e) Adjusted predictions using 11×11 neighboring windows for aggregation and calibration. LMA = 88.63%. (f) Adjusted predictions using 17×17 neighboring windows for aggregation and calibration. LMA = 88.88%. (g) Adjusted predictions using 21×21 neighboring windows for aggregation and calibration. LMA = 88.72%. (e) Adjusted predictions using 25×25 neighboring windows for aggregation and calibration. LMA = 89.15%.


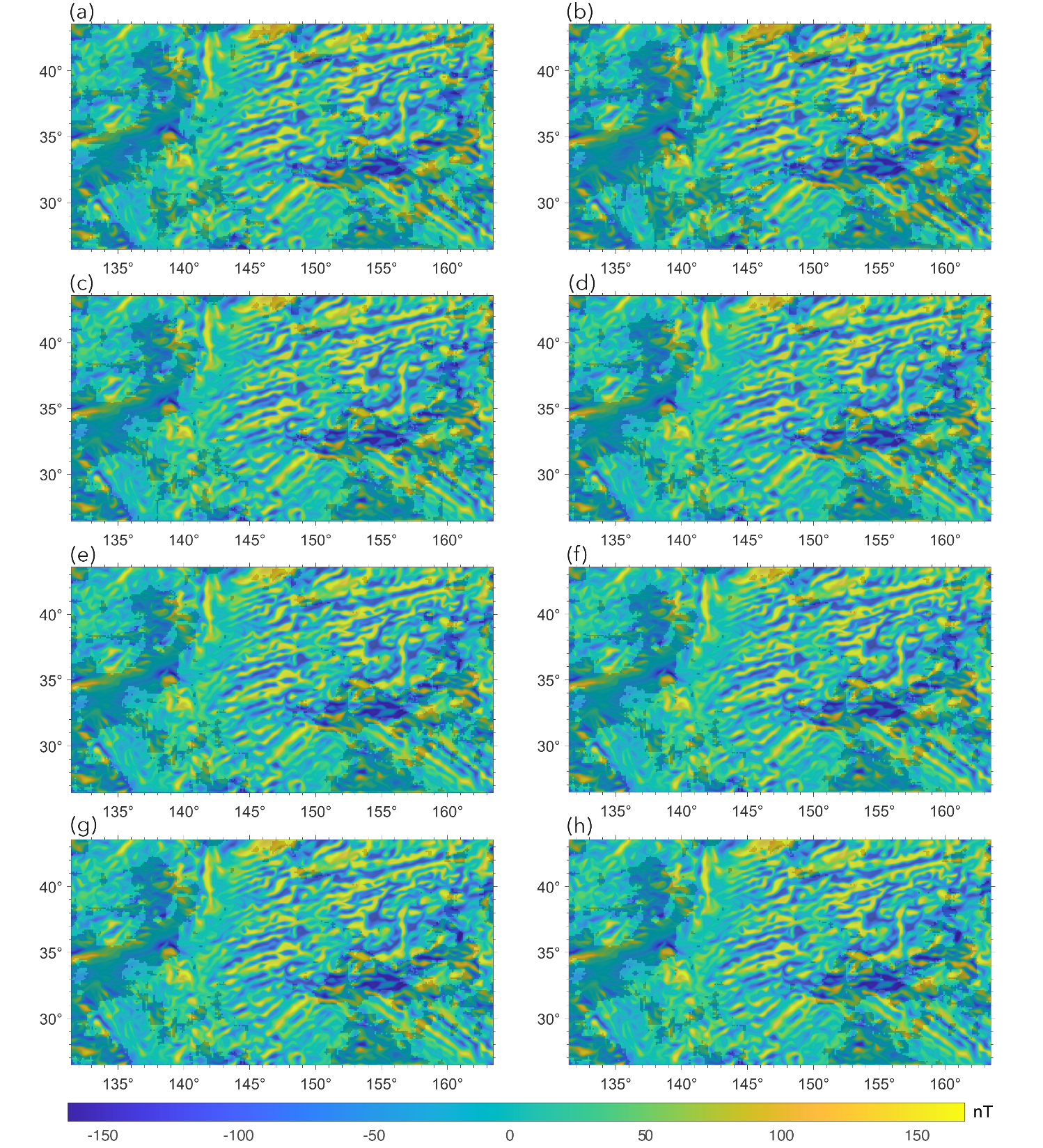


**Figure S3:** Prediction maps for magnetic anomalies at Shatsky Rise when different cutoff values and various sets of neighboring windows were used. (a) Raw predictions when a cutoff value of 0.5 is used. No aggregation and calibration are applied. LMA = 71.3%. (b) Raw predictions when a cutoff value of 0.7 is used. No aggregation and calibration are applied. LMA = 58.87%. (c) Adjusted predictions using 3×3 neighboring windows for aggregation and calibration. LMA = 72.47%. (d) Adjusted predictions using 5×5 neighboring windows for aggregation and calibration. LMA = 73.20%. (e) Adjusted predictions using 11×11 neighboring windows for aggregation and calibration. LMA = 73.74%. (f) Adjusted predictions using 17×17 neighboring windows for aggregation and calibration. LMA = 73.99%. (g) Adjusted predictions using 21×21 neighboring windows for aggregation and calibration. LMA = 74.21%. (e) Adjusted predictions using 25×25 neighboring windows for aggregation and calibration. LMA = 74.54%.
